# The optimal time of initiation of renal replacement therapy in acute kidney injury: A meta-analysis

**DOI:** 10.18632/oncotarget.17946

**Published:** 2017-05-16

**Authors:** Kaiping Luo, Shufang Fu, Weidong Fang, Gaosi Xu

**Affiliations:** ^1^ Medical Center of the Graduate School, Nanchang University, Nanchang, China; ^2^ Department of Nephrology, People's Hospital of Ganzhou, Ganzhou, China; ^3^ Department of Nephrology, The Second Affiliated Hospital of Nanchang University, Nanchang, China

**Keywords:** acute kidney injury, renal replacement therapy, mortality, meta-analysis

## Abstract

**Background:**

The impact on the timing of renal replacement therapy (RRT) initiation on clinical outcomes for patients with acute kidney injury (AKI) remains controversial.

**Materials and methods:**

We searched the Cochrane Library, EMBASE, Global Health, MEDLINE, PubMed, the International Clinical Trials Registry Platform, and Web of Science.

**Results:**

We included 49 studies involving 9698 patients. Pooled analysis of 5408 critically ill patients with AKI showed that early RRT was significantly associated with reduced mortality compared to late RRT [odds ratio (OR), 0.40; 95% confidential intervals (CI), 0.32 - 0.48; *I^2^*, 50.2%]. For 4290 non-critically ill patients with AKI, there was no statistically significant difference in the risk of mortality between early and late RRT (OR, 1.07; 95% CI, 0.79 - 1.45; *I^2^*, 73.0%). Early RRT was markedly associated with shortened intensive care units (ICU) length of stay (LOS) and hospital LOS compared to late RRT in both critically ill and non-critically ill patients with AKI.

**Conclusions:**

Early RRT probably reduce the mortality, ICU and hospital LOS in critically ill patients with AKI. Inversely, early RRT in non-critically ill patients with AKI did not decrease the mortality, but shortened the ICU and hospital LOS.

## INTRODUCTION

Acute kidney injury (AKI) is increasingly common and associated with adverse clinical outcomes, including excess mortality and morbidity, and prolonged hospital length of stay (LOS) [1–4]. Renal replacement therapy (RRT) is the cornerstone for the treatment of severe AKI. Although RRT provokes a considerable escalation in the complexity of therapy, the optimal timing of initiation of RRT in patients with AKI has been the focus of those debates [5, 6]. Conflicting results from clinical trials and systematic reviews have not resolved the debates, leaving clinicians to select the timing of initiation of RRT based on suboptimal evidence.

Studies aimed at determining the optimal time for starting RRT have evaluated the various arbitrary cut-offs for time from Intensive Care Unit (ICU) admission [7–9] or development of a biochemical “start time” [10, 11], AKI stage [12, 13], serum urea [14, 15], urine output [16, 17], fluid balance [18], and serum creatinine [15, 19, 20]. However, the arbitrary cut-offs often differentiated between early and late RRT. Some data suggested that early compared with late RRT reduced the mortality with better renal recovery. Early initiation of RRT may produce benefits by avoiding hypervolemia, eliminating of uremic toxins, establishing acid-base homeostasis, and preventing other complications such as gastric hemorrhage and metabolic encephalopathy [7, 13, 16]. Late RRT may allow time for the stabilization of a patient’s condition before RRT and may even avoid the RRT [12, 21–23]. Gaudry et al. showed that the mortality was lower in patients who never received RRT than those received RRT early or late (37.1% vs. 48.5% or 61.8%), and the patients with late RRT were the most severely ill at baseline [13]. Thus, we hypothesized that the different severity of illness for patients with AKI who received early RRT may produce distinct effects on mortality. Therefore, we firstly performed a meta-analysis according to the severity of illness for patients with AKI to investigate the opportunity of RRT initiation.

3 earlier meta-analyses (Seabra et al. [24] identified 23 studies, Karvellas et al. [25] identified 15 studies and Wang et al. [26] included 51 trials) showed that early RRT could confer a survival benefit. 11 trials performed before 1985 in Seabra et al. and Wang et al. were excluded, and the addition of 10 recently published studies have been included in the present meta-analysis. However, a recent meta-analysis found no significant difference in mortality between early and late RRT [27], but included only nine “high-quality” studies. Furthermore, the included studies were limited with high heterogeneity. In the present study, we firstly made a definition of early RRT based on time-based cutoffs for patients with AKI to investigate the optimal timing of initiation of RRT.

## RESULTS

### Study enrolment and characteristics

**Figure 1:**
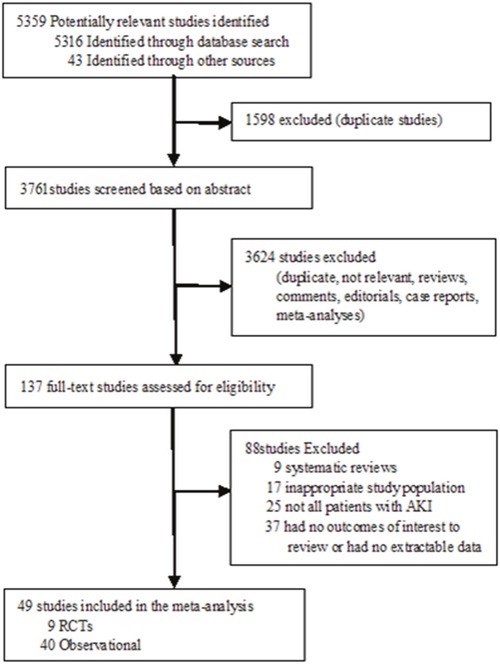
Flow diagram for the selection of studies inclusion in the meta-analysis

**Figure 2:**
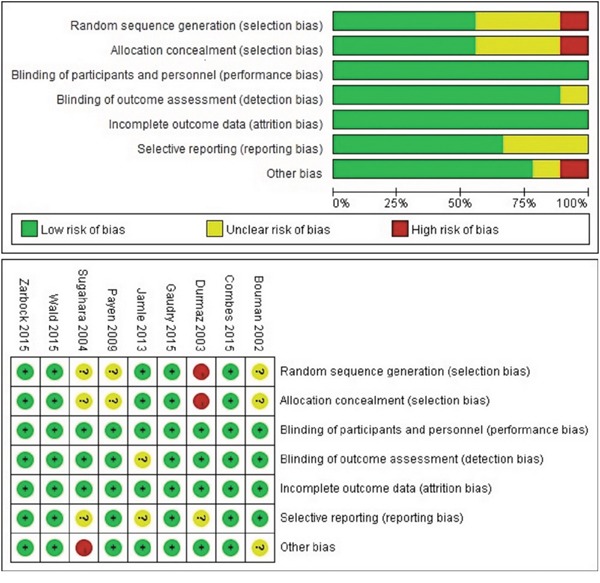
Risk of bias summary of early versus late RRT initiation on mortality in patients with AKI on randomized controlled trial

Figure 1 outlines the process for study selection. 49 studies including 9 RCTs [10, 12, 13, 15, 16, 19, 21–23] and 40 observational studies [7–9, 11, 14, 17, 18, 20, 28–59] were included in our meta-analysis. The eligible studies were conducted from 1985 to 2016 with 9698 patients evaluated the timing of initiation of RRT in patients with AKI. The characteristics of the articles were listed in Table 1, and the details of risk of bias for RCTs were showed in Figure 2.

**Table 1:** The fundamental characteristics and patient demographic data of included studies reporting data on early RRT versus late RRT

Auther, Year	Country	Study Design	Population	Early Mortality	Late Mortality	Severity ofIllness	Early RRT Criteria	Late RRT Criteria	Quality
Early time to RRT <12 h									
Bouman2002	Netherlands	RCT	Multisystem	20/70	9/36	Early: SOFA 10.3;Late: SOFA 10.6	Time to RRT<12 h	Time to RRT>12h	M
Piccinni2006	Italy	Retrospective	Sepsis; ICU	18/40	29/40	Early: APACHE2=27.2;Late: APACHE2=27.8	Time to RRT <12 h	No RRT	7
Andrade2007	Brazil	Retrospective	Multisystem;Leptospirosis	3/18	10/15	Early: APACHE2=24.5;Late: APACHE2=26	Mean time to RRT = 4.4hrs	Mean time to RRT = 27.3hrs	5
Wu VC2007	China	Retrospective	Acute LiverFailure;Surgical ICU	34/54	22/26	Early: APACHE2=18;Late: APACHE2=19	Mean time from ICU admit to RRT =4.4hrs; BUN<80 mg/dL ANDtraditional indications present	Mean time from ICU admit to RRT =11.1hrs; BUN>80 mg/dL ANDtraditional indications present	6
Manche2008	Malta	Retrospective	Post CardiacSurgery	14/56	13/15	NR	Mean RRT start 8.6hrs post-op; Oliguria unresponsive to med mgmt	Mean RRT start 41.2hrs post-op; Oliguria refractory to med mgmt	6
Ji2011	China	Retrospective	Post CardiacSurgery	3/34	9/24	Early: APACHE3= 69;Late: APACHE3= 88.2p<0.001	Time from urine output <0.5ml/kg/h to RRT <12h; Mean oliguria to start of RRT 8.4hrs	Time from urine output <0.5ml/kg/h to RRT >12h; Mean oliguria to start of RRT21.5hrs	6
Shum2013	China	Retrospective	Multisystem;Sepsis	43/89	15/31	Early: SOFA 13;Late: SOFA 12P=0.011	Mean time from ICU admit to RRT= 10.8hrs (RIFLE criteria:‘Injury’ or ‘Failure’ criteria)	Mean time from ICU admit to RRT =20.7hrs (RIFLE criteria:‘pre- Risk’ or ‘Risk’ criteria)	6
Serpytis2014	Lithuania	Retrospective	Multisystem;Sepsis	30/42	39/43	NR	Time from anuria to RRT <12hrs	Time from anuria to RRT >12hrs	5
Wald2015	Canada	RCT	Multisystem	16/48	19/52	Early: SOFA 13.3;Late: SOFA 12.8	Mean time to RRT = 9.7hrs	Meantime to RRT = 32hrs;Classic indications for RRT	H
Crescenzi2015	Italy	Prospective	Post CardiacSurgery	28/46	10/13	NR	Time from urine output <0.5ml/kg/hto RRT <12h	Time from urine output <0.5ml/kg/h to RRT >12h	6
Zarbock2015	Germany	RCT	Multisystem	44/112	65/119	Early: SOFA 15.6;Late: SOFA 16.0	Time to RRT <8h; KDIGO stage 2	Time to RRT <12h; Stage 3 AKIor no initiation	H
Gaudry2015	France	RCT	Multisystem	150/311	153/308	Early: SOFA 10.9;Late: SOFA 10.8	Time to RRT <6h; Stage 3 AKI	Classic indications for RRT; Oliguria or anuria >72hrs after randomization	H
Early time to RRT <24 h									
Elahi2004	UK	Retrospective	Post Cardiacsurgery	8/36	12/28	NR	Mean RRT start 0.78 days;Low urine output <100ml within 8h after surgery	Mean RRT start 2.5 days; Traditional indications: Urea≥30mmol/L, Cr ≥250mmol/L, K >6.0mEq/L	6
Demirkilic2004	Turkey	Retrospective	Post CardiacSurgery	8/34	15/27	NR	Mean RRT start 0.88 days;Low urine output <100ml within 8hrs post-op;	Mean RRT start 2.56 days;Cr ≥5mg/dL, or K >5.5 mEq/L	6
Boussekey2012	France	Retrospective	Multisystem	28/67	28/43	Early: SOFA: 11.1;Late: SOFA 8.8;p=0.002	Time from RIFLE- ‘Injury’ to RRT< 16hrs; Mean time to RRT=6hrs	Time from RIFLE- ‘Injury’ to RRT > 16hrs; Mean time to RRT=64hrs	7
Chon2012	Korea	Retrospective	Multisystem;Sepsis	7/36	9/19	Early: SOFA 13.5;Late: SOFA 12	Time to RIFLE ‘Injury’/‘Failure’< 24hrs; Mean time to RRT=12.5hrs	Time to RIFLE ‘Injury’/‘Failure’> 24hrs; Mean time to RRT= 42.2hrs	7
Leite2013	Brazil	Retrospective	Multisystem	33/64	67/86	Early: APACHE2=19.2;Late: APACHE2=18.7	Time from AKIN 3 diagnosis to RRT <24hrs	Time from AKIN 3 diagnosis to RRT >24hrs	7
Jun2014	Australia	Prospective	Multisystem;Sepsis	82/219	84/220	Early: SOFA: 2.0;Late: SOFA 2.1	Time from AKI diagnosis to RRT <17.6hrs	Time from AKI diagnosis to RRT>17.6hrs	6
Combes2015	France	RCT	Post CardiacSurgery	40/112	40/112	Early: SOFA 11.5;Late: SOFA 12.0	RRT initiated <24hrs and continuedfor min of 48hrs	Traditional indications for RRT	H
Yang2016	China	Retrospective	Post CardiacSurgery	20/59	80/154	Early: APACHE2=21.4.;Late: APACHE2=23.1	AKI in absence of traditional indications for RRT; persistence of hypotension (for more than 6 h) despite preload optimization;	Traditional indications for RRT	7
Early time to RRT <48 h									
Durmaz2003	Turkey	RCT	Post CardiacSurgery	1/21	7/23	NR	Cr rise >10% from pre-op levelwithin 48hrsof surgery	Cr rise >50%from pre-op level;or Urine output <400ml/24hrs	L
Lyem2009	Turkey	Prospective	Post CardiacSurgery	5/95	6/90	NR	Low urine output triggering RRT started <48hrs; Evidence of 50% increase in BUN,	Time >48hrs to start of RRT for similar markers of renal failure managed medically for minimum 48hrs	7
Bagshaw2009	Multicountries	Prospective	Multisystem	462/785	304/442	Early: SOFA 10.9;Late: SOFA 10.7p=0.04	RRT started <2d from ICU admission	RRT started >2d from ICU admission	7
Perez2012	Spain	Prospective	MultisystemSepsis	71/135	78/109	Early: SOFA 12;Late: SOFA 11	Time from ICU admission to RRT < 48h	Time from ICU admission to RRT > 48h	5
Lim2014	Singapore	Prospective	Multisystem	37/56	36/84	Early: SOFA 11;Late: SOFA 7;p=0.001	RRT started < 2d from admission;Traditional indications for RRT	RRT started > 2d from admission; AKIN stage 1 or 2 with indication or AKIN stage3	6
Hyung2016	Korea	Retrospective	MultisystemSepsis	9/30	17/30	Early: APACHE2=22.9;Late: APACHE2=21.1	Time to RRT <26.4 h	Time to RRT >26.4 h	6
Early time to RRT <72 h									
Sugahara2004	Japan	RCT	Post CardiacSurgery	12/14	2/14	Early: APACHE2=18;Late: APACHE2=19	Mean time to RRT start 1.7d±0.8 post op; UOP <20ml/hrs ×2hrs + OR UOP <500ml/day	Mean time to RRT start 18d±0.9 post op; UOP <30ml/hrs ×3hrs ORUOP <750ml/day	L
Sabater2009	Spain	Prospective	Multisystem	21/44	68/104	Early: APACHE2=26;Late: APACHE2=24	Mean RRT start 2.2d post ICU admit (RIFLE criteria: RISK & INJURY)	Mean RRT start 6.4d post ICU admit (RIFLE criteria: FAILURE)	7
Fernandez2011	Spain	Retrospective	Post CardiacSurgery	59/111	74/92	NR	RRT started <3d after cardiac surgery	RRT started >3d after cardiac surgery	5
Shiao2012	China	Retrospective	Surgical	236/436	143/212	Early: SOFA 11.4;Late: SOFA 11.3	Time to development of traditional RRT indications <3d; Mean time to start of RRT 1.4d	Traditional RRT indications AND start of RRT >3 d; Mean time to start of RRT 18d	6
Early time to RRT >72 h									
Gettings1999	USA	Retrospective	Multisystem;Trauma	25/41	47/59	Early ISS = 33.0;Late ISS = 37.2	Mean RRT start post admission10d; BUN <60mg/dl AND Oliguria, Vol overload, Electrolytes, Uremia;	Mean RRT start post admission 19d; BUN >60 mg/dL AND Oliguria, Electrolytes, Uremia;	5
Shiao2009	China	Prospective	MajorAbdominalSurgery	22/51	34/47	Early: SOFA 8.3;Late: SOFA 8.5	Mean Time to RRT from ICU Admit =7.3d (RIFLE criteria:RISK or pre-RISK criteria)	Mean Time to RRT from ICU Admit = 8.4d (RIFLE criteria:INJURY or FAILURE criteria)	7
Chung2009	US	Retrospective	Severe BurnedPatients	9/29	24/28	Early: SOFA 13;Late: SOFA 13	Mean time from admit to RRT =17 days; AKIN stage2(+shock)/3	Mean time from admit to AKIN stage 2(+shock)/3 but not dialyzed = 23 days	6
Carl2010	US	Retrospective	Multisystem;Sepsis	44/85	42/62	Early: APACHE2=24.8;Late: APACHE2=24.7	Mean ICU stay prior to RRT = 6.3d;BUN <100mg/dL + AKIN stage >2;	Mean ICU stay prior to RRT = 12.3d; BUN > 100mg/dL + AKIN stage >2;	7
Hyung2012	Korea	Retrospective	Multisystem	75/105	81/105	Early: SOFA 14.4;Late: SOFA 14.4	Time from ICU admission to RRT =4.7d	Time from ICU admission to RRT =4.8d	7
RRT initiated base on biochemical indicators; Meantime to initiation of RRT not specified									
Kresse1999	Germany	Retrospective	Multisystem	83/141	102/128	NR	BUN≤34mmol/L, sCr 380umol/L, and urine output 924 ml/24h	BUN >34mmol/L, sCr 477umol/L, and urine output 525 ml/24h	7
Splendiani2001	Italy	Retrospective	Multisystem	6/14	3/13	NR	BUN≤ 33mmol/L	BUN> 59 mmol/L and/or severe electrolyte disturbances	5
Tsai2005	China	Retrospective	Multisystem	42/67	30/31	NR	BUN< 29 mmol/L	BUN> 29 mmol/L	5
Liu2006	Multicountries	Prospective	Multisystem	43/122	50/121	NR	Azotemia defined by BUN < 76mg/dL	Azotemia defined by BUN > 76mg/dL	6
Payen2009	France	RCT	Multisystem	20/37	17/39	Early: SOFA 11.6;Late: SOFA 10.4	RRT × 96hrs w/diagnosis of ‘sepsis’	No RRT; unless metabolic renal failure & classic indications for RRT present	M
Elsevivrs2010	Belgium	Prospective	Multisystem	379/653	280/650	Early: SOFA 9.9;Late: SOFA 8.5p=0.001	Serum Cr >2mg/dL	No RRT	5
Konopka2011	Poland	Retrospective	Multisystem	17/25	11/12	NR	As soon as AKI was diagnosed	After full treatment for HF and unsuccessful pharmacological treatment of complicating AKI	5
Chou2011	China	Retrospective	Sepsis;Surgery ICU	135/192	124/178	Early: SOFA 10.8;Late: SOFA 11.6	RIFLE criteria: RISK or pre-RISK	RIFLE criteria: INJURY or FAILURE	6
Nascimento2012	Brazil	Retrospective	Multisystem	9/23	43/63	Early: APACHE 2= 21;Late: APACHE 2= 28	BUN ≤26.7 mmol/L	BUN>26.7 mmol/L	6
Wu SC2012	China	Retrospective	MultisystemSurgery	10/20	45/53	Early: SOFA 9.5;Late: SOFA 10.0	RIFLE criteria: RISK	RIFLE criteria: INJURY or FAILURE	5
Hu2013	China	Retrospective	Multisystem	20//36	8/13	Early: SOFA 9.3;Late: SOFA 11.5	AKIN 1and 2 (Cr >200-300%baseline &Urine<0.5cc/kg/h for >12h)	AKIN 3 (Cr ≥354μmol/L or Cr >300% baseline & urine <0.3cc/kg/h for 24h or anuria >12h)	5
Jamle2013	India	RCT	Multisystem	21/102	13/106	Early: SOFA 7.3;Late: SOFA 8.2	Cr >618μmol/L	Traditional indications for RRT	M
Gaudry2014	France	Retrospective	Multisystem;Sepsis	44/91	29/112	Early: SOFA 9;Late: SOFA 8P<0.01	RRT criteria: Cr ≥300μmol/L, Urea >25mmol/L, K >6.5mmol/L,pH <7.2, Oliguria, Vol overload,	No RRT	5
Tian(46^1^)2014	China	Retrospective	Multisystem;Sepsis	5/23	11/26	Early: SOFA 7.6;Late: SOFA 8.4	AKIN 1 (Cr ≥26.4μmol/L or >150- 200% baseline & urine < 0.5cc/kg/h for >6h)	No RRT	6
Tian(46^2^)2014	China	Retrospective	Multisystem;Sepsis	12/31	14/21	Early: SOFA 9.3;Late: SOFA 9.6	AKIN 2 (Cr >200-300% baseline &Urine <0.5cc/kg/h for >12h)	No RRT	6
Tian(46^3^)2014	China	Retrospective	Multisystem;Sepsis	31/46	11/13	Early: SOFA 10;Late: SOFA 11.2	AKIN 3 (Cr ≥354μmol/L or Cr >300% baseline & urine < 0.3cc/kg/h for 24h or anuria >12h)	No RRT	6

LEGEN: AKI Acute kidney injury, RRT renal replacement therapy, Cr Creatinine, UOP Urine output, ICU Intensive Care Unit, AKIN Acute Kidney Injury Network, RIFLE Risk, Injury, Failure, Loss and End-stage, KDIGO Kidney Disease: Improving Global Outcomes, RCTs randomized clinical trials, Quality Score: The Cochrane Collaboration Risk of Bias tool for RCTs and Newcastle-Ottawa Scale for observational studies, H High quality: low risk of bias, M Medium quality: unclear risk of bias, L Low quality: high risk of bias, APACHE Acute Physiology and Chronic Health Evaluation, SOFA Sequential Organ Failure Assessment, NR Not reported.

### Meta-analysis results

#### Primary outcomes

**Figure 3:**
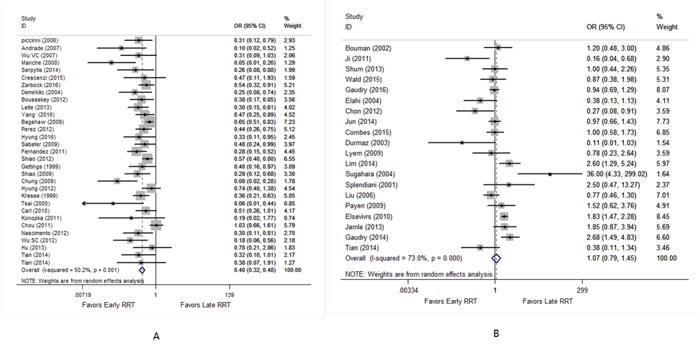
Forest plot shows the effect of early versus late RRT on mortality in critically ill **(A)** and non-critically ill patients with AKI **(B)**.

Pooled analysis of 5408 critically ill patients with AKI showed that early RRT was markedly associated with reduced mortality compared to late RRT (OR, 0.40; 95% CI, 0.32 - 0.48; *I^2^*, 50.2%, Figure 3). For 4290 non-critically ill patients with AKI, there was no statistically significant difference in the risk of mortality between early and late RRT (OR, 1.07; 95% CI, 0.79 - 1.45; *I^2^*, 73.0%, Figure 3).

Subgroup analysis of critically ill patients was firstly conducted in the present study by using the definition of early according to time criteria versus biochemical indicators. The significant association between early RRT and reduced mortality was also found under the studies that defined early by time criteria [early RRT within 12 hours (OR, 0.28; 95% CI, 0.16 - 0.49; *I^2^*, 44.8%), within 24 hours (OR, 0.37; 95% CI, 0.25 - 0.54; *I^2^*, 0.0%), within 48 hours (OR, 0.55; 95% CI, 0.39 - 0.77; *I^2^*, 30.8%), within 72 hours (OR, 0.45; 95% CI, 0.29 - 0.69; *I^2^*, 48.2%), and after 72 hours (OR, 0.32; 95% CI, 0.14 - 0.74; *I^2^*, 71.4%)], and by biochemical parameters (OR, 0.40; 95% CI, 0.25 - 0.64; *I^2^*, 58.9%). Subgroup analysis of non-critically ill patients depending on the definition of early showed no significant subgroup survival benefits from early RRT.

Subgroup analysis of critically ill patients was based on the type of ICU admission. Early RRT was significantly associated with reduced mortality compared to late RRT among surgical group (OR, 0.33; 95% CI, 0.22 - 0.48; *I^2^*, 47.9%) and mixed group (OR, 0.43; 95% CI, 0.34 - 0.54; *I^2^*, 49.8%). Subgroup analysis of non-critically ill patients based on ICU admission type showed no evidence of survival advantage in early RRT.

Subgroup analysis of critically ill patients was also performed according to RRT modality [continuous renal replacement therapy (CRRT), intermittent hemodialysis (IHD) or Mixed]. We found a markedly significant reduce in mortality in critically ill patients assigned to early RRT in the CRRT group (OR, 0.40; 95% CI, 0.30 - 0.54; *I^2^*, 28.4%), IHD group (OR, 0.11; 95% CI, 0.03 - 0.43; *I^2^*, 56.9%) and Mixed group (OR, 0.45; 95% CI, 0.35 - 0.57; *I^2^*, 53.6%) when compared to late RRT. Subgroup analysis of non-critically ill patients according to RRT modality showed that early RRT could not confer a survival benefit (Table 2).

**Table 2:** Outcomes measures of early versus late RRT initiation

Outcome or Subgroup	Group A: critically ill patients with AKI					Group B: non-critically ill patients with AKI				
	Studies	No. of Patients	Study Reference No	Effect Estimate (95% CI)	*p*	Studies	No. of Patients	Study Reference No	Effect Estimate (95% CI)	*p*
Primary Outcomes: early versus late RRT initiation on mortality										
All studies	31	5408	7-9,12,18,28-30,32,34,35,38-41,43,44, 46^2^,46^3^,47,48,50-59	OR, 0.40 (0.32 to 0.48)	0.001	20	4290	10,11,13-17,19-23,31,33,36,37,42,45,46^1^,49	OR, 1.07 (0.79 to 1.45)	0.000
Subgroup stratified by the definition of early according to time criteria and biochemical indicators on mortality										
Time: Early RRT <12h	7	639	9,12,28-30,32,56	OR, 0.28 (0.16 to 0.49)	0.093	5	1003	10,13,21,31,42	OR, 0.86 (0.58 to 1.29)	0.201
Time: Early RRT <24h	4	534	34,35,53,54	OR, 0.37 (0.25 to 0.54)	0.691	4	782	11,22,33,36	OR, 0.72 (0.43 to 1.19)	0.097
Time: Early RRT <48h	3	1531	7,55,57	OR, 0.55 (0.39 to 0.77)	0.236	3	368	17,19,37	OR, 0.82 (0.18 to 3.79)	0.012
Time: Early RRT <72h	3	999	18,38,58	OR, 0.45 (0.29 to 0.69)	0.145	1	28	16	OR, 36.0 (4.33 to 299.02)	NE
Time: Early RRT >72h	4	465	8,39,40,52	OR, 0.32 (0.14 to 0.74)	0.015	0	NE	NE	NE	NE
Biochemicl indicators	10	1240	41,43,44, 46^2^,46^3^-48,50,51,59	OR, 0.40 (0.25 to 0.64)	0.009	7	2109	14,15,20,23,45, 46^1^,49	OR, 1.46 (0.96 to 2.23)	0.008
Subgroup stratified by surgical versus mixed medical admissions on mortality										
Surgical	9	1506	8,9,18,30,32,34,38,44,54	OR, 0.33 (0.22 to 0.48)	0.053	6	602	16,17,19,22,31,33	OR, 0.71 (0.24 to 2.07)	0.000
Mixed medical	22	3902	7,12,28,29,35,39,41,43,46^2^,46^3^-48,50-53,55-59	OR, 0.43 (0.34 to 0.54)	0.004	14	3688	10,11,13-15,20,21,23,36,37,42,45,46^1^,49	OR, 1.22 (0.91 to 1.63)	0.000
Subgroup stratified by RRT modality on mortality										
Mixed	14	3442	7,9,12,28,29,35,38,41,43,48,53,54,55,57	OR, 0.45 (0.35 to 0.57)	0.009	6	2495	13,14,20,21,45,49	OR, 1.32 (0.86 to 2.03)	0.000
CRRT	14	1771	8,18,32,34,39,40,44,46^2^,46^3^,47,50,52,55,58	OR, 0.40 (0.30 to 0.54)	0.152	12	1544	10,11,15-17,22,31,33,36,37,42, 46^1^	OR, 0.92 (0.58 to 1.46)	0.017
IHD	3	255	30,51,59	OR, 0.11 (0.03 to 0.43)	0.098	2	251	19,23	OR, 0.56 (0.04 to 8.73)	0.000
Secondary outcomes: ICU and Hospital LOS										
ICU LOS	8	862	28,34,35,38,41, 46^2^,46^3^,53	MD, −0.41 (−0.55 to −0.27)	0.000	4	336	17,19,31, 46^1^	MD, −1.47 (−1.71 to −1.22)	0.000
Hospital LOS	6	755	8,28,34,38,39,54	MD, −0.36 (−0.51 to −0.21)	0.000	3	287	17,19,31	MD, −1.07 (−1.31 to −0.82)	0.415

LEGEN: OR odds ratio, 95% CI confidence interval, *P* Test for Heterogeneity, MD mean difference, RRT renal replacement therapy, ICU Intensive Care Unit, CRRT continuous renal replacement therapy, IHD intermittent hemodialysis, Mixed CRRT and/or IHD and/or other RRT modality, LOS length of stay, NE not evaluable.

#### Secondary outcomes

For critically ill patients with AKI, as showed in Table 2, early RRT significantly shortened ICU (MD, −0.41; 95% CI, −0.55 to −0.27; *I^2^*, 87.0%) and hospital LOS (MD, −0.36; 95% CI, −0.51 to −0.20; *I^2^*, 94.7%) compared to late RRT. Similar results were obtained in non-critically ill patients with AKI in ICU (MD, −1.47; 95% CI, −1.71 to −1.22; *I^2^*, 89.3%) and hospital LOS (MD, −1.07; 95% CI, −1.31 to −0.82; *I^2^*, 0%).

#### Sensitivity, meta-regression analyses

**Figure 4:**
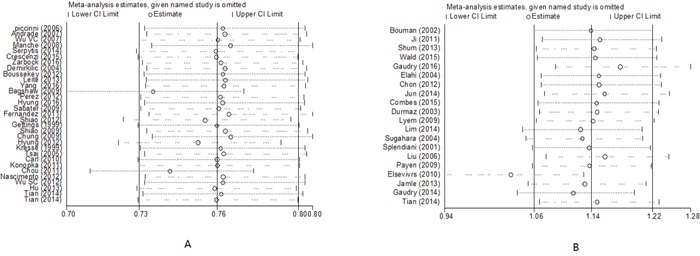
Sensitivity analyses of early versus late RRT on mortality in critically ill **(A)** and non-critically ill patients with AKI **(B)**.

Statistically similar results were obtained after omitting each study of critically ill patients with AKI, and the results of the sensitivity analyses were robust. Sensitivity analyses showed that Elsevivrs et al. [20] was the main source of heterogeneity for the studies of non-critically ill patients with AKI, and the heterogeneity was significantly decreased by omitting the study. For non-critically ill patients with AKI, there was no statistically significant difference in the risk of mortality between early and late RRT with the study (OR, 1.07; 95% CI, 0.79 - 1.45; *I^2^*, 73.0%) or without the study (OR, 1.02; 95% CI, 0.74-1.40; *I^2^*, 66.8%). Elsevivrs et al. was a large sample trial with 1303 patients when compared to other articles including not more than 619 subjects (Figure 4).

With the meta-regression, we did not find a correlation between patient mortality and study design (RCT *vs.* observational), RRT modality (CRRT, IHD *vs.* Mixed), study quality score, severity of illness [Sequential Organ Failure Assessment (SOFA) score], ICU admission type (surgical *vs.* mixed medical admissions). However, we find a correlation between patient mortality and sample size (n ≥ 100 *vs.* n < 100, *P* = 0.001) in critically ill patients with AKI.

#### Publication bias

**Figure 5:**
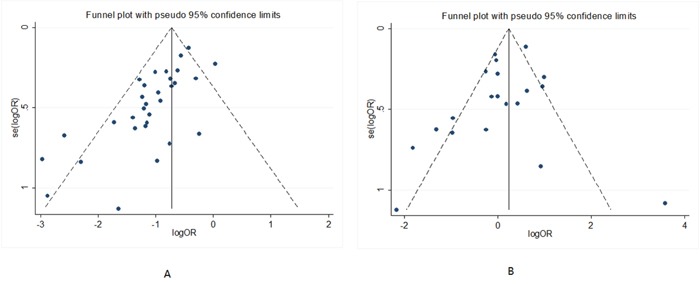
Begg’s funnel plots of early versus late RRT on mortality in critically ill **(A)** and non-critically ill patients with AKI **(B)**.

No potential publication bias was observed in non-critically ill patients with AKI (*P* = 0.347 for the Begg test, and *P* = 0.169 for the Egger test). Publication bias was seen in critically ill patients with AKI (*P* = 0.001 for the Begg test, and *P* = 0.000 for the Egger test, Figure 5).

## DISCUSSION

We identified 49 studies reported data on the timing of RRT initiation among 9698 patients with AKI, and we found that early RRT significantly reduced the mortality compared to late RRT in critically ill patients with AKI. In addition, no significant survival benefits associated with early RRT were seen in non-critically ill patients with AKI. Early RRT was markedly associated with shortened ICU and hospital LOS compared to late RRT in both critically ill and non-critically ill patients with AKI.

Regardless of the definition of early RRT (according to time criteria or biochemical indicators), ICU admission type (surgical *vs.* mixed) or RRT modality (CRRT, IHD *vs.* Mixed), subgroup analyses of critically ill patients with AKI did reveal survival benefits from early RRT. Furthermore, subgroup analyses of non-critically ill patients with AKI showed that no evidence of survival advantage in early RRT.

In the present study, we firstly performed the meta-analysis according to the severity of illness and definition of early RRT based on time-based cutoffs for patients with AKI to investigate the time of RRT initiation. We accepted a broad definition of “critically ill patients with AKI” based on AKI with multiple-organ dysfunction syndrome [60], septic shock [40], RIFLE criteria (failure, loss of function, and end-stage kidney disease) [37, 43, 44], AKIN stages 3 [41, 42, 46] or Kidney Disease: Improving Global Outcomes (KDIGO) stage 3 [12, 61].

By the meta-regression, we found sample size was one of the sources of heterogeneity. In contrast to previous meta-analyses, we found a lower heterogeneity among studies on this topic, especially in the subgroup. We noted those critically ill patients in early RRT within 12 hours (*I^2^*, 44.8%), 24 hours (*I^2^*, 0.0%), 48 hours (*I^2^*, 30.8%), and 72 hours (*I^2^*, 48.2%) showed the lower heterogeneities, indicating that the heterogeneity may be partially explained by the definition of early RRT timing. However, we could not account for the observed heterogeneity by meta-regression according to study design, RRT modality, the study quality score, severity of the illness, and ICU admission type. Thereby, the heterogeneity observed is most likely explained by the differences in definitions for early RRT timing, the inability to account for heterogeneity in clinical practice and critical care patterns, many confounding factors that affect the mortality, publication bias, sample size and the inclusion of retrospective, prospective and RCTs.

The present systematic review has some limitations. Firstly, definitions for AKI are to some extent different in the included studies. Secondly, the definition of early RRT based on various arbitrary cut-offs for time, which ultimately downgraded the strength of evidence. Thirdly, there were publication bias and significant heterogeneity in the present study. Many confounding factors affect the mortality, and meta-regression may not be enough to verify this issue. Lastly, the association with mortality is largely dependent on observational studies and might have been affected by allocation or selection bias. Thus, further high-quality RCTs focused on mortality according to the optimal time for starting RRT are necessary to fully understand the effects of early RRT for patients with AKI.

## MATERIALS AND METHODS

### Participants, interventions and outcome measures

We included studies that evaluated the timing of initiation of RRT in patients with AKI. For the review, early and late RRT were defined based on criteria used by the authors in their studies. early and late RRT were defined as extended time-based cutoffs (arbitrary cut-offs for time from ICU admission or development of a biochemical “start time”), or biochemical indicators [serum creatinine, serum urea, RIFLE (risk, injury, failure, loss of function, and end-stage kidney disease) classifications, Acute Kidney Injury Network (AKIN) stages, urine output, and fluid balance]. Late RRT criteria also included conventional RRT indications (hyperkalemia, acidosis or fluid overload) and expectant care (no RRT initiated). The primary outcome was mortality, and the secondary outcomes were ICU and hospital LOS.

### Searching strategies

We searched the Cochrane Library, EMBASE, Global Health, MEDLINE, PubMed, the International Clinical Trials Registry Platform, and Web of Science from January 1985 to November 2016. Owing to a low likelihood of relevance to modern RRT and critical care practices, studies published before 1985 were excluded in the present study. Keywords include acute renal failure/acute kidney injury/renal insufficiency, mortality, renal replacement therapy/renal dialysis/hemodialysis/dialysis. The related research references were also reviewed.

### Inclusion and exclusion criteria

The inclusion criteria were as follows: (1) randomized clinical trials (RCTs) and/or observational cohort studies; (2) studies evaluating the timing of initiation of RRT in patients with AKI with direct effect on mortality; (3) complete data available to calculate odds ratio (OR) or mean difference (MD) with 95% confidence interval (CI); (4) clear definitions of AKI stated. Exclusion criteria were as follows: (1) data from the studies could not be extracted and analyzed; (2) duplicate publications; (3) non-human experimental studies.

### Study selection and data extraction

Two investigators (Kaiping Luo and Shufang Fu) independently performed the study selection. All the disagreements were resolved by discussion. Data extraction included first author, year of publication, country, study design, sample size, age, sex, RRT modality, mortality, ICU LOS, hospital LOS, and definitions of early and late RRT.

Dr. Gaudry and colleagues [13] showed that the mortality was lower in the patients who never received RRT than those received RRT early or late. Patients who received RRT late were the most severely ill at baseline, and patients who never received it were less ill at baseline. More than 50% mortality in critically ill patients with AKI received RRT was confirmed by many randomized controlled trials [1, 3, 4, 60]]. Thus, we hypothesized that critically ill patients with AKI who receive early RRT may decrease mortality, non-critically ill patients with AKI may confer survival benefits without early RRT. Subjects were identified as being of “critically ill patients” if the late RRT group with high mortality rates (≥ 50%), or “non-critically ill patients” if the late RRT group with low mortality rates (< 50%).

### Quality assessment

The Cochrane Collaboration Risk of Bias tool was used to assess RCTs [62]. This tool consists of 6 domains and assesses 5 specific biases. A judgment of low risk, unclear risk, or high risk was provided for each domain. The Newcastle-Ottawa Scale (NOS) was used in the assessment of quality of cohort studies [63]. NOS quality assessment scale ranges from 0 to 9 stars. The star evaluates 3 main categories: selection, comparability, and outcome.

### Statistical analysis

Statistical analysis was performed using Review Manager (version 5.3) and STATA statistical software (version 12.0). We calculated OR with 95% CI for dichotomous data and MD with 95% CI for continuous data. Statistical heterogeneity of the data was quantified using the *I^2^* test, and the *I^2^*> 50% indicated significant statistical heterogeneity. Sensitivity analysis, meta-regression analyses and subgroup analysis were conducted to investigate the potential sources of heterogeneity. Publication bias was assessed by constructing a funnel plot and using the Egger regression test and the Begg rank correlation test. A *P* value less than 0.05 was considered statistically significant.

## CONCLUSIONS

Our data suggest that early RRT probably reduce the mortality, ICU and hospital LOS in critically ill patients with AKI. Inversely, early RRT in non-critically ill patients with AKI did not decrease the mortality, but shorted the ICU and hospital LOS.

## Abbreviations

RRT: renal replacement therapy
AKI: acute kidney injury
OR: odds ratio
CI: confidential intervals
ICU: intensive care units
LOS: length of stay
RIFLE: risk, injury, failure, loss of function, and end-stage kidney disease
AKIN: Acute Kidney Injury Network
RCTs: randomized clinical trials
MD: mean difference
NOS: Newcastle-Ottawa Scale
CRRT: continuous renal replacement therapy
IHD: intermittent hemodialysis
SOFA: Sequential Organ Failure Assessment
KDIGO: Kidney Disease: Improving Global Outcomes
