# Correction: The optimal time of initiation of renal replacement therapy in acute kidney injury: A meta-analysis

**DOI:** 10.18632/oncotarget.24254

**Published:** 2018-01-15

**Authors:** Kaiping Luo, Shufang Fu, Weidong Fang, Gaosi Xu

**Affiliations:** ^1^ Department of Nephrology, The Second Affiliated Hospital of Nanchang University, Nanchang, China; ^2^ Department of Nephrology, People’s Hospital of Ganzhou, Ganzhou, China

This article has been corrected: Dr. Xu citation has been changed to affiliation 1 and affiliation 3 is now deleted.

Original article: Oncotarget. 2017; 8:68795-68808. https://doi.org/10.18632/oncotarget.17946

